# Case report of emergency repair of injury to the great vessels of the clavicular region by coated endovascular stent implantation

**DOI:** 10.3892/etm.2013.1097

**Published:** 2013-05-02

**Authors:** JIAN WANG, JIE LAO, YANGBO LIU, GAOBAO ZHUO, HUAIBAO ZHANG, LEI CHEN

**Affiliations:** 1 First Affiliated Hospital of Wenzhou Medical College, Wenzhou, Zhejiang 325000;; 2 Shanghai Huashan Hospital, Fudan University, Shanghai 200040, P.R. China

**Keywords:** combined injury, axillary artery, coated endovascular stent, neurolysis

## Abstract

The subclavian artery leaves the thoracic cage at the outer margin of the first rib, where it becomes the axillary artery. Rupture and hemorrhage of the subclavian artery may result in ischemia and necrosis of the upper limb, brachial plexus injury, and even hemorrhagic shock or mortality. A patient with an injury to the proximal segment of the axillary artery underwent emergency repair using a coated endovascular stent graft. The patient was followed up for 13 months and examined using CT imaging, B-mode ultrasonography and electromyography to evaluate stent function and brachial plexus recovery. The endovascular stent graft remained correctly positioned and patent, extending across the injured part of the vessel. Neurolysis at 3 months after injury was effective in restoring the majority of the brachial plexus function. The coated endovascular stent graft was effective in treating the acute injury to the great vessels in the clavicular region. Follow-up of brachial plexus function is important and secondary neurolysis should be performed if necessary.

## Introduction

The majority of injuries to the great vessels in the clavicular region are caused by penetrating trauma, which often results in uncontrollable hemorrhage and brachial plexus injury. Recently, coated endovascular stent grafting has been used to treat injuries to the great vessels in the clavicular region (1,2), and balloon-expandable and self-expandable endovascular intervention has successfully treated patients with false aneurysms in these vessels following injury (3,4). However, these methods have certain limitations. In the present study the patient presented with an injury to the proximal segment of the axillary artery complicated by brachial plexus injury and was treated with emergency coated endovascular stent placement under C-arm fluoroscopy guidance on July 31, 2010. The patient was followed up for 13 months and the results were favorable.

## Case report

**Figure 1.**
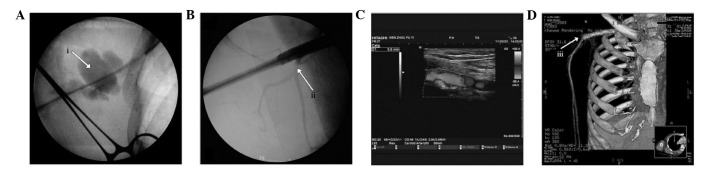
(A) CT scan of the injured region. Arrow i: contrast medium leaked from the proximal segment of the right axillary artery and the distal vessels were not visualized. (B) C-arm fluoroscopy-guided intraoperative arteriography. Arrow ii: the contrast medium passed normally through the proximal axillary artery and the distal and collateral vessels were clearly visualized. (C) B-mode ultrasonography at 6 months after surgery. The stent was patent, with no thrombus and slight intimal hyperplasia (2.7 mm) was present at the widest part of the inner wall. Blood flow was slightly reduced compared with the contralateral side. (D) CT arteriography at 6 months after surgery. Arrow iii: CT arteriography with three-dimensional reconstruction and distal vessel reconstruction, showing that the stent was correctly positioned and patent, with no distortion or deformation.

A 31-year-old male was injured by baamboo and was admitted to Fuding People’s Hospital (Fuding, China) for wound debridement and suturing. However, the patient was transferred to The Affiliated Hospital of Wenzhou Medical College (Wenzhou, China) due to progressive swelling and numbness of the right chest wall and absence of pulses in the right upper limb. Physical examination showed that the patient was anemic, with a reduced level of consciousness, clear breath sounds bilaterally and blood pressure <70/40 mmHg in the left upper limb following volume expansion. Oxygen saturation of the right upper limb was 0% and the pulse rate was 110 bpm. There were two right thoracic wounds; a 2-cm wound located 1.5 cm below the medial end of the clavicle, and a 3-cm wound located below the lateral end of the clavicle in the anterior axillary fossa. The wounds were sutured, with evident capillary hemorrhage in the wound at the lateral end of the clavicle. The right chest wall and the right upper limb were swollen. The right axillary, brachial and ulnar pulses were absent, and there was no capillary return in the fingers. Traction on the limb produced pain and increased numbness. Muscle strength was grade II and muscle tone was normal. Thoracic CT scan images are shown in Fig. 1. The patient was diagnosed with hemorrhagic shock, right axillary artery injury, right brachial plexus injury, right scapular fracture, damage to the right shoulder muscles and a large right shoulder hematoma. This study was approved by the ethics committee of First Affiliated Hospital of Wenzhou Medical College. The informed consent was obtained from the patient.

Color ultrasonography showed an injury at the junction of the right subclavian artery and the axillary artery. Emergency repair of the injury was performed under general anesthesia with tracheal intubation. A longitudinal incision was made on the medial aspect of the right upper arm and an endovascular stent delivery catheter system was inserted into the brachial artery. C-arm fluoroscopy-guided arteriography showed leakage of contrast medium from the proximal segment of the axillary artery and the distal vessels were not visualized (Fig. 1A). A 5-cm Wallgraft artificial coated endovascular stent with Unistep Plus propulsion system (Boston Scientific Ireland Ltd., Galway, Ireland) was placed in the injured vessel. Arteriography following stent placement showed contrast medium passing normally through the proximal axillary artery, with distal and collateral vessels clearly visualized (Fig. 1B). Radial and ulnar pulses were palpable following the procedure, but limb swelling increased following the intervention, possibly due to ischemia/reperfusion injury and venous injury. Considering the signs of brachial plexus injury, an exploratory surgery was performed immediately. During the surgery, exploration revealed a false aneurysm in the proximal segment of the axillary artery. Following removal of the hematoma, a 1.5-cm U-shaped wound was covered with a coated endovascular stent. Gauze was used to stop bleeding and the ruptured accompanying vein was ligated. There was no disruption of the brachial plexus and the surrounding hematoma was removed. Clopidogrel was administrated orally for 2 weeks to inhibit platelet aggregation postoperatively.

One week after injury, a CT scan of the right clavicular region showed that the stent was correctly positioned and patent, with no surrounding false aneurysm. A second-look surgery was performed to remove the gauze and the organized blood clot surrounding the stent. Doppler ultrasonography (Fig. 1C) and CT arteriography (CTA; Fig. 1D) were performed at 1, 3 and 6 months after the second-look surgery, and showed a patent coated endovascular stent, normal blood flow wave pattern, and the right upper limb with 97–100% oxygen saturation on finger pulse oximetry.

**Figure 2.**
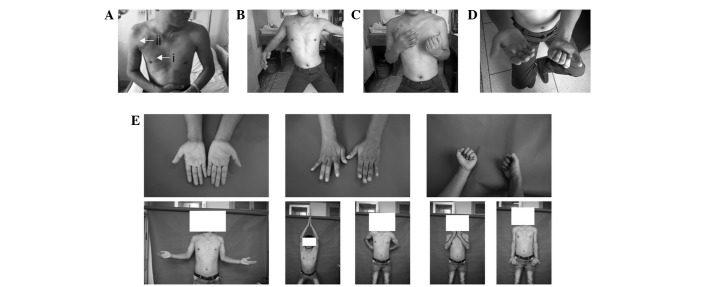
(A) One month after surgery. Arrow i: pectoralis major and pectoralis minor atrophy; arrow ii: deltoid muscle atrophy. (B–D) Shoulder, elbow, wrist and hand function at 1 month after injury: Right shoulder abduction is reduced to 40°, elbow flexion is weak, and the patient is not able to flex the wrist or fingers. (E) Upper limb functional recovery at 12 months after injury. All upper limb functions were restored except for intrinsic muscle function of the hand.

**Figure 3.**
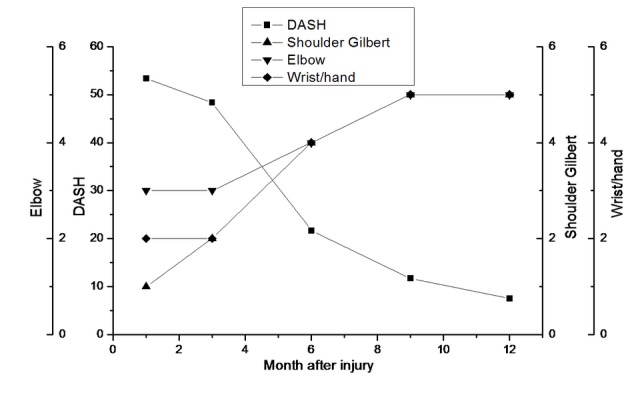
Shoulder, elbow, wrist and hand functional scores, and disabilities of the arm, shoulder and hand (DASH) scores. From 1 to 3 months after injury, elbow and wrist/hand function remained impaired, the shoulder regained some function, and the DASH scores were reduced. After the second neurolysis (3.5 months after injury), the shoulder, elbow and wrist/hand functional scores increased, and the DASH scores further decreased.

After injury, traction on the right upper limb produced pain and increased numbness, and muscle strength was grade II. During the first surgery, a false aneurysm was found in the proximal segment of the axillary artery, with surrounding hematoma causing brachial plexus compression. The hematoma was removed to decompress the brachial plexus. One week after the initial surgery, a second-look surgery was performed to examine the brachial plexus and remove the organized blood clot. At 1 month after the initial operation, the patient had developed atrophy of the pectoralis major, pectoralis minor, deltoid and infraspinatus muscles (Fig. 2A). Right upper limb function was examined (Fig. 2B–D) and evaluated using various clinical scales; the Gilbert score (5) for shoulder joint function was stage I, the Gilbert and Raimondi score (5) for elbow function was 3 points (Grade II), the Raimondi score (5) for hand and wrist function was stage II and the disabilities of the arm, shoulder and hand (DASH) score (6) was 53.33 (Fig. 3). After 3 months of oral neurotrophic medication, the Gilbert score for shoulder function had increased to stage II and the DASH score had decreased to 48.33, indicating partial recovery of shoulder function. However, the elbow, hand and wrist function scores had not improved, and the region innervated by the ulnar nerve had not recovered. Electromyography (EMG) showed injury to the right cord of the brachial plexus, including severe injury to the median, ulnar and axillary nerves and mild injury to the radial and musculocutaneous nerves. The second brachial plexus neurolysis was performed at 3.5 months after injury. The right upper limb function was re-evaluated following the second brachial plexus neurolysis, and the shoulder, elbow, wrist and hand function and DASH scores are shown in Fig. 2E and Fig. 3.

The patient was followed up until 13 months postoperatively. Color B-mode Doppler ultrasonography showed that the stent at the junction of the right axillary artery and the subclavian artery had a 5.3–8.7 mm internal diameter, with intimal thickening of ≤2.7 mm, and slower blood flow than that on the contralateral side. Part of the right axillary vein was reversed and used to form a collateral branch. CT angiography (CTA) showed the stent and the normal size and appearance of the right subclavian and axillary arteries, with no evidence of stenosis. These results indicate that the endovascular stent was stable and remained patent *in vivo*, and may be used to repair injured great vessels in the clavicular region. Notably, stent distortion or deformation due to the large range of motion of the shoulder joint did not cause stenosis during the follow-up period. However, the possibility of stenosis resulting from the large range of motion should be considered when repairing vessels close to the shoulder joint. All parameters of EMG at 13 months were significantly improved compared with the results at 3 months. The right upper limb function was almost restored to normal, with the exception of hypothenar muscle atrophy, limited interphalangeal joint extension, limited intrinsic muscle function and numbness of the fourth and fifth fingers and ulnar palm. The DASH score at 13 months was 7.5, indicating minimal influence on the life and work of the patient.

## Discussion

Since the subclavian artery is protected by the clavicle and the superficial muscles, the incidence of subclavian artery injury is extremely low (7). However, rupture of the subclavian artery causes local hemorrhage and may result in ischemia and necrosis of the upper limb, brachial plexus injury and even hemorrhagic shock or mortality. In the present study, the arterial injury was in the proximal segment of the axillary artery, at the point where the subclavian artery exits the thoracic cage, and was repaired using techniques for subclavian artery repair. Subclavian and proximal axillary artery injuries are usually repaired early to promptly reconstruct blood flow (1,2,6). Since the wall of the subclavian artery is very elastic, loosening of the arterial clamp during vascular anastomosis may lead to the proximal end of the subclavian artery retracting into the thoracic cavity, possibly resulting in increased hemorrhage (8). Vascular anastomosis is also more difficult following hemorrhage, and the incidence rate of thrombosis and distal vascular embolism is increased (9).

In the present study, we placed the coated endovascular stent under C-arm fluoroscopy guidance rather than using a digital subtraction angiography workstation, as it was simpler and more convenient. Patients with suspected arterial and venous injury or brachial plexus injury may be treated with intravascular stent grafting followed by open surgery. Simultaneously, the veins and the brachial plexus may be examined to determine the necessity for anastomosis and repair, thus reducing the concern over further hemorrhage. However, the surgeon should handle the tissues gently to avoid damaging the implanted stent. Coated endovascular stent grafting may effectively stop bleeding and greatly reduce surgical risks (10). Interventional surgeries have previously been performed under digital subtraction angiography (DSA) guidance (4), which is simple and obtains high quality images, but is not appropriate for patients with severe injuries to the great vessels.

Coated endovascular stent grafts are not recommended for use in limbs since the large range of motion of the limbs may cause the stents to distort or deform. In the present case, the injury was located in the proximal segment of the axillary artery, where the range of motion is limited. At the 13 month follow-up, CTA showed no obvious stent displacement or distortion. Coated endovascular stent grafting is therefore safe for this type of injury. However, further investigation is required to determine the safety of using this technique for injuries adjacent to the shoulder joint.

Intimal thickening, stenosis and embolism are common complications following endovascular stent placement, and directly influence postoperative function and prognosis. The coated endovascular stent has a smoother inner wall than other endovascular stents, which reduces the incidence of intimal thickening, stenosis and embolism. The patency of coated endovascular stents has been reported as 98.2, 89.5 and 84.5% at 1, 2 and 5 years postoperatively (8). In the present case, a 5-cm endovascular stent was placed and there was intimal thickening after 6 months which was not increased at 13 months. A study with a larger sample size and a longer follow-up period should be undertaken to further evaluate the long-term prognosis of such stents.

Since it is difficult to determine clinically if brachial plexus injury is a direct result of nerve injury or is secondary to vessel injury, exploratory surgery is important in cases of subclavian artery injury, even when there is no severe limb ischemia.

Following treatment of injury to the great vessels, upper limb function should be evaluated and brachial plexus injury should be treated. It is important to determine the necessity and timing of secondary surgery. Patients with brachial plexus injury show certain disturbances of upper limb function, which may recover following treatment. It is generally accepted that there may be partial spontaneous recovery of nerve function prior to surgery. Dubuisson and Kline (11) suggest that a second-look surgery should be performed at 2–4 months after open brachial plexus injury if there has been no recovery of nerve function. However, other researchers have different opinions on the optimal timing of second-look surgery, ranging from 1 to 6 months (12,13). One previous study observed that a delay of longer than 5 months may result in poor long-term upper limb function (14).

Magalon *et al* (15) stated that exploration should be performed within 7 days after the injury since exploration is easier at that time and allows earlier nerve grafting. In the present case, the hematoma was removed and complete or partial rupture of the brachial plexus was prevented. Therefore, the timing of the second surgery depended on the neurological recovery of the patient. At the 3 month follow-up, the function of the elbow, wrist and hand had not recovered significantly, and shoulder function had improved slightly. EMG results indicated the necessity for a second surgery. The subsequent recovery of upper limb function demonstrated the effectiveness of the second neurolysis.

C-arm fluoroscopy-guided endovascular stent grafting shortens treatment time and facilitates treatment for brachial plexus injury and other complications. Open trauma to the infraclavicular region is complicated by brachial plexus injury. Therefore, early exploration to assess nerve continuity, removal of the surrounding hematoma, active follow-up, regular examination of stent location and patency, evaluation of brachial plexus function and optimal timing of the second neurolysis operation may help to restore upper limb function.
